# Robo-Therm, a pipeline to RNA thermometer discovery and validation

**DOI:** 10.1261/rna.079980.124

**Published:** 2024-07

**Authors:** Davis M. Sharts, Maria T. Almanza, Andrea V. Banks, Alyssa M. Castellanos, Catherine G.O. Hernandez, Monica L. Lopez, Daniela Rodriguez, Alina Y. Tong, Maximilian R. Segeberg, Luiz F.M. Passalacqua, Michael M. Abdelsayed

**Affiliations:** 1 Department of Biology, California Lutheran University, Thousand Oaks, California 91360, USA; 2 Laboratory of Nucleic Acids, National Heart, Lung, and Blood Institute, National Institutes of Health, Bethesda, Maryland 20892, USA

**Keywords:** bioinformatics, gene regulation, microbiology, noncoding RNA, RNA thermometer

## Abstract

RNA thermometers are highly structured noncoding RNAs located in the 5′-untranslated regions (UTRs) of genes that regulate expression by undergoing conformational changes in response to temperature. The discovery of RNA thermometers through bioinformatics is difficult because there is little sequence conservation among their structural elements. Thus, the abundance of these thermosensitive regulatory structures remains unclear. Herein, to advance the discovery and validation of RNA thermometers, we developed Robo-Therm, a pipeline that combines an adaptive and user-friendly in silico motif search with a well-established reporter system. Through our application of Robo-Therm, we discovered two novel RNA thermometers in bacterial and bacteriophage genomes found in the human gut. One of these thermometers is present in the 5′-UTR of a gene that codes for *σ*^*70*^ RNA polymerase subunit in the bacteria *Mediterraneibacter gnavus* and *Bacteroides pectinophilus,* and in the bacteriophage Caudoviricetes, which infects *B. pectinophilus*. The other thermometer is in the 5′-UTR of a tetracycline resistance gene (*tetR*) in the intestinal bacteria *Escherichia coli* and *Shigella flexneri*. Our Robo-Therm pipeline can be applied to discover multiple RNA thermometers across various genomes.

## INTRODUCTION

Recent discoveries have revealed that many bacteria respond to heat stress through temperature-sensing RNAs that use structure-based strategies to regulate gene expression (Narberhaus et al. 2006; Meyer et al. 2017). RNA thermometers are highly structured RNA elements existing in the 5′-untranslated regions (UTRs) of mRNAs (Altuvia et al. 1989; Loh et al. 2018; Sharma et al. 2022). In most characterized examples, RNA thermometers increase gene expression of a downstream gene in response to higher temperatures (Kortmann and Narberhaus 2012; Tong et al. 2023). Most RNA thermometers function by folding into a stable secondary structure at physiologically low temperatures (<30°C) to sequester the Shine–Dalgarno (SD) sequence in a stem, preventing the ribosome from accessing the SD. Conversely, when they encounter physiologically higher temperatures (up to 45°C), the increased thermodynamic energy denatures the secondary structure of the RNA thermometer, exposing the SD sequence to allow ribosomal binding and initiation of translation, resulting in increased expression of the downstream gene (Smith et al. 2010; Loh et al. 2018).

To date, there are two classes of well-characterized and conserved RNA thermometers that share common secondary structures and motifs. FourU thermometers contain one to four hairpins, with the terminal hairpin having at least four consecutive uracil nucleotides base-pairing with the SD sequence (Waldminghaus et al. 2007b; Noll et al. 2021). The fourU motif typically contains at least two noncanonical G•U wobble base pairs (Waldminghaus et al. 2007b; Noll et al. 2021; Tong et al. 2023). These noncanonical base pairs contribute to the destabilization of the terminal hairpin upon heat stress; this causes the hairpin to melt and denature in a zipper-like mechanism (Kortmann and Narberhaus 2012). The second highly conserved class of thermometers, ROSE-like (repression of heat shock gene expression) thermometers, contain two to four hairpins, with the terminal hairpin containing the SD sequence imperfectly base-paired with a highly conserved U(U/C)GCU motif. The ROSE-like motif typically contains a predicted bulged G and noncanonical base-pairing (Waldminghaus et al. 2009; Abduljalil 2018). The bulged G and noncanonical base-pairing in this motif denote its temperature sensitivity due to this network of weak hydrogen bonds, allowing the SD-containing hairpin to open when exposed to increased temperature (Chowdhury et al. 2006). Many other reported RNA thermometers do not contain the same motifs as the fourU and ROSE-like thermometers and have yet to be grouped into specific classifications (Scheller et al. 2021).

Several RNA thermometers have been discovered through transcriptome probing or by bioinformatic predictions. Both methods exploit the secondary structures of RNA thermometers to identify potential candidates. Transcriptome-wide probing marries structure probing techniques with next-generation sequencing (NGS) to identify genes that are differentially expressed in a specific organism during heat stress. Transcriptome probing has led to the discovery of several RNA thermometers in *Yersinia pseudotuberculosis* (Righetti et al. 2016) and RNA thermometers that regulate the translation of glycerol permease genes in *Bacillus subtilis* (Jolley et al. 2023). Transcriptome probing can reveal the abundance of many RNA thermometers at once but is usually constrained to one organism.

Bioinformatics has been used to identify novel RNA thermometers across multiple genomes (Narberhaus et al. 2006; Waldminghaus et al. 2007a; Chursov et al. 2013; Churkin et al. 2014; Tong et al. 2023). These approaches emphasize predicted secondary structure searching due to higher conservation of secondary structure rather than sequence identity among RNA thermometers. RNAthermsw (Churkin et al. 2014) and RNAtips (Chursov et al. 2013) both use RNAfold (Hofacker 2003), an in silico RNA structure prediction software, and temperature simulations to discover candidate thermometer sequences by predicted changes in energy. RNA-SURIBA (Waldminghaus et al. 2007a) is a database that uses Mfold, another in silico RNA structure predication software (Zuker 2003), to identify new candidate RNA thermometers. RNA-SURIBA uses Mfold to create bracket annotations to define previously annotated thermometers as a template to find new candidates. Additionally, well-defined motifs of RNA thermometers can be used as scaffolds to search for functional RNAs with similar predicted structures and function in programs such as MSARI (Coventry et al. 2004), CMfinder (Yao et al. 2006), Infernal (Nawrocki and Eddy 2013), and RNArobo (Rampášek et al. 2016).

**FIGURE 1.**
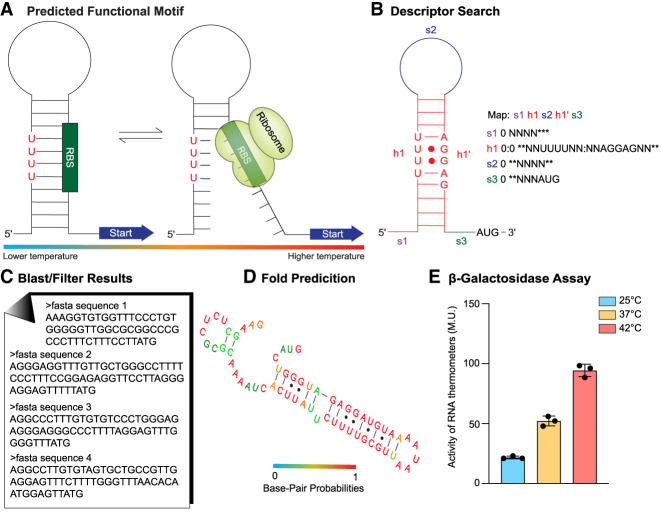
Robo-Therm pipeline for RNA thermometer discovery and validation. (*A*) The characterized structure and predicted functional motif of a validated RNA thermometer are used as a scaffold to search for similar RNA thermometers. (*B*) RNArobo search consisting of a map and descriptor based on the characterized secondary structure is performed, with “s” denoting single-stranded regions, “h” denoting helical regions in the structure, “N” denoting any nucleotide, and “*” denoting a nucleotide may or may not be present. (*C*) RNArobo results are submitted to NCBI Blast and filtered to select candidate thermometers. (*D*) The secondary structure of the candidate thermometers is predicted via RNAfold (Hofacker 2003). (*E*) Example of β-galactosidase assay of cells after incubation at 25°C, 37°C, and 42°C used to determine the RNA thermometer activity.

Herein, we developed a bioinformatic-based pipeline, termed Robo-Therm, for the discovery of novel RNA thermometers across various genomes (Fig. 1). Through this pipeline, we have previously identified and validated a fourU RNA thermometer (Tong et al. 2023), and here we report two novel fourU RNA thermometers that occur across different prokaryotic genomes. The first occurs upstream of a tetracycline resistance gene (*tetR*) in the intestinal bacteria *Escherichia coli* and *Shigella flexneri*. The second discovered RNA thermometer occurs upstream of the RNA polymerase *σ*^*70*^ subunit in the bacteria *Mediterraneibacter gnavus* and *Bacteroides pectinophilus* as well as a bacteriophage Caudoviricetes, which infects *B. pectinophilus* (Liang and Bushman 2021; Gulyaeva et al. 2022). This is our second example of an RNA thermometer discovered in a bacteriophage and its host.

## RESULTS

### Using bioinformatics to identify candidate RNA thermometer sequences

Robo-Therm uses the RNA motif descriptor program RNArobo (Rampášek et al. 2016) to search through annotated genomes for RNA thermometers. Most impactful RNA motif prediction programs commonly use covariance models, a probabilistic approach using primary sequence and secondary structure (Coventry et al. 2004; Yao et al. 2006; Nawrocki and Eddy 2013). RNARobo allows users to input customized descriptors to narrow and refine searches for RNA structures through any genomic data set in FASTA format. We used RNArobo because of its flexibility in sequence input, completely defined by the user, allowing researchers to fully handcraft and feature components of an RNA that are believed to be crucial to its function. RNArobo, or its predecessor RNAbob, has been used to discover ribozymes in various genomes, and we recently used it to discover the *blyA* fourU RNA thermometer that occurs in the genomes of the SPβ prophage and its host, *B. subtilis* 168 (Webb et al. 2009; Webb and Lupták 2018; Tong et al. 2023). RNArobo is uniquely amenable to the discovery of RNAs that are more conserved in secondary structure than sequence, making it an efficient tool for RNA thermometer discovery.

**FIGURE 2.**
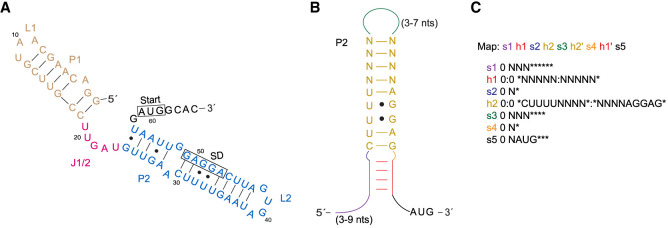
Using RNArobo to search for potential RNA thermometers similar to the second stem of the *agsA* thermometer. (*A*) The entire *agsA* RNA thermometer secondary structure (Waldminghaus et al. 2007b). (*B*) A visualization of the map and descriptor. (*C*) RNArobo search using the second stem (P2 and L2) of the *agsA* thermometer as a scaffold. Our map and descriptor with the conservation of four uracil nucleotides in a hairpin base paired with the Shine–Dalgarno sequence, and ending with the AUG start codon. “s” denotes single-stranded regions, “h” denotes helical regions in the structure, “N” denotes any nucleotide, and “*” denotes a nucleotide may or may not be present.

**FIGURE 3.**
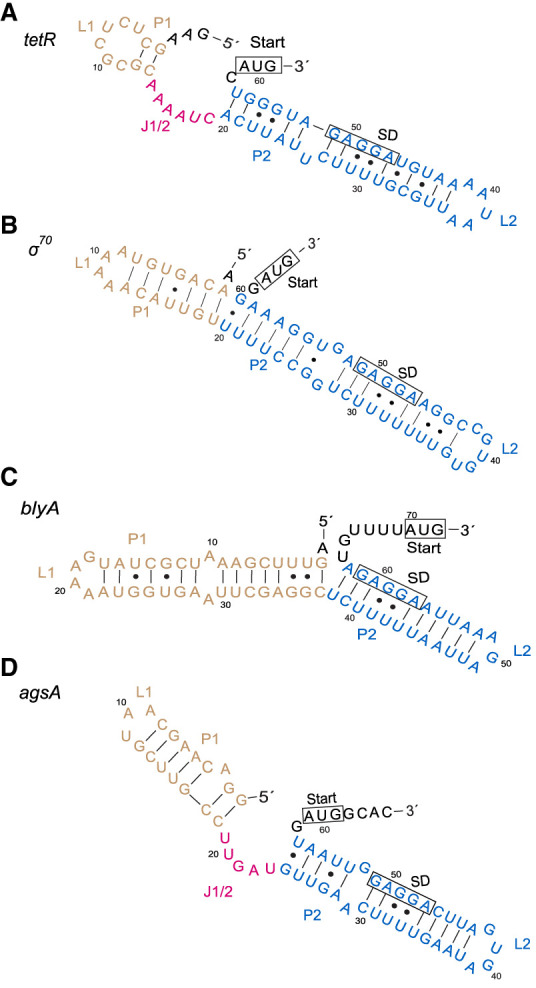
Secondary structure prediction of the *tetR* (*A*), *σ*^*70*^ (*B*), *blyA* (*C*), and *agsA* (*D*) RNA thermometers. The start codons for their respective downstream genes and the SD sequences are boxed and labeled. Stems in the structure are labeled with “P,” single-stranded loops are labeled with “L,” and a single-stranded region linking two stems is labeled “J1/2” to indicate the junction of stems one and two.

To search for new potential RNA thermometers, previously characterized RNA thermometer motifs (Fig. 1A) were used as the template to develop new RNA maps and descriptors (Fig. 1B). Despite the presence of defined core motifs in fourU and ROSE-like thermometers, the overall secondary structure and sequences of thermometers containing these motifs can vary significantly, resulting in unique search outcomes for each template RNA explored. Furthermore, searches of each specific RNA thermometer can vary, depending on which features, such as insertions and mismatches, are defined by the user and how strict or permissive those features are defined. We chose to identify potential fourU RNA thermometers based on the predicted secondary structure of the extensively studied *agsA* fourU thermometer (Figs. 2A and 3D; Waldminghaus et al. 2007b). The *agsA* thermometer stands out as an exemplary template because of the abundance of literature discussing its structure and function (Waldminghaus et al. 2007b; Shah and Gilchrist 2010; Meyer et al. 2017). Moreover, we successfully used the *agsA* thermometer as a scaffold in the identification of the similarly structured *blyA* thermometer (Tong et al. 2023).

Sequences from our RNArobo search results were curated and filtered through NCBI Blast (Fig. 1C) and ranked according to proximity to a gene, to secondary structure predictions in RNAfold (Fig. 1D; Hofacker 2003), and to the function of the downstream gene. Predicted structures of candidates are sorted based on possessing defined motif features that are essential for thermometer function. For example, a promising candidate for a fourU thermometer should contain four consecutive uracil nucleotides base-paired with an SD sequence. Generally, candidates that are identified as directly in the 5′-UTR of an annotated gene are chosen. These searches can be applied to unannotated genomes as well (such as soil metadata sets), to study the prevalence of thermometers in certain environments. The predicted SD should occur 5–10 nt upstream of the start codon of the annotated gene of interest, to ensure the necessary distance for ribosome initiation (Steitz and Jakes 1975). Candidates from our search results are fused to a thermally stable β-galactosidase reporter (Hirata et al. 1984; Klinkert et al. 2012) to test and validate thermometer function (Fig. 1E). From our search results, we tested two new potential fourU thermometers with predicted secondary structures that contain the fourU motif and that occur in genomes that are found in the human gut microbiome (Table 1; Supplemental Table S1).

**TABLE 1.** Genomic description of RNA thermometers discovered by Robo-Therm

Gene	Protein description	Genome
*tetR*	*tetR* family transcriptional regulator	*Escherichia coli* *Shigella flexneri*
*σ* ^*70*^	RNA polymerase subunit *σ*^*70*^	*Mediterraneibacter gnavus* *Bacteroides pectinophilus* Caudoviricetes (bacteriophage)
*blyA*	Autolysin enzyme (*N*-acetylmuramoyl-l-alanine amidase)	*Bacillus subtilis* SPβc2 (bacteriophage)

RNArobo searches consist of a map that lists each structural element of the secondary structure and a descriptor that defines each component of the map (Rampášek et al. 2016). Single-stranded (s) and paired elements (h) were manually described for each search (Figs. 1B and 2A,B). It is important to include elements that are essential for thermometer function such as the SD and start codon. Other elements to be considered are those specific to the template that the search is based on. For example, for the *agsA*-based search, we included four uracils across from the SD (Fig. 2B). For our search, we used the second hairpin of the *agsA* thermometer as a scaffold for our map and descriptor to find similar RNA structures containing a fourU motif (Fig. 2C). Incorporating highly conserved elements unique to a class of RNA thermometers led to the discovery of new thermometers in that class.

The first candidate fourU thermometer was found in the 5′-UTR of a *tetR/acrR* family transcriptional regulator (*tetR*) (Fig. 3A; Supplemental Fig. S1A), with a 100% sequence identity match in *E. coli* and *S. flexneri.* The TetR-family of transcriptional regulators (TFTRs) play an important role in antimicrobial resistance, as regulators of antibiotic efflux pumps (Cuthbertson and Nodwell 2013; Colclough et al. 2019). The second fourU candidate was found in the 5′-UTR of the RNA polymerase subunit *σ*^*70*^ (Fig. 3B; Supplemental Fig. S1B), with a 100% sequence identity match in the bacteria genomes of *M. gnavus*, *B. pectinophilus*, and the bacteriophage Caudoviricetes (Caudoviricetes sp. isolate ctoMw15). *σ*^*70*^ is a family of primary initiation factors in bacteria for the RNA polymerase complex and directs the RNA polymerase to specific promoter sites of a wide variety of genes (Paget and Helmann 2003). Interestingly, Caudoviricetes is one of the most abundant groups of phages found in the human gut and infects bacteria of the phylum Bacteriodete (Liang and Bushman 2021; Gulyaeva et al. 2022). Although the impact of heat stress on the gut microbiome remains limited, it is known that heat stress increases bacterial translocation, infection, and increased risk of septic shock in humans (Schwab et al. 2014; Ogden et al. 2020; Huus and Ley 2021).

*σ*^*70*^ is known as a “housekeeping” factor because of its critical role in mediating the transcription of many essential genes (Gruber and Gross 2003). Multiple members of the *σ*^*70*^ family can occur in a single genome, including up to 63 in a single bacterial genome (Paget and Helmann 2003). Interestingly, both *M. gnavus* and *B. pectinophilus* contain several paralogs of *σ*^*70*^, suggesting that *σ*^*70*^ may not be widely regulated by heat stress in these genomes. The presence of the *σ*^*70*^ thermometer and gene in the bacteriophage Caudoviricetes may suggest that regulation by heat is mediated by the life cycle of the bacteriophage; however, there is no current evidence to make this direct correlation.

Our results were curated from a single descriptor and search based on the second hairpin of the *agsA* thermometer. The predicted secondary structures of the candidates *tetR*, *σ*^*70*^, and our previously characterized *blyA* thermometer all contain structurally similar terminal hairpins with the *agsA* thermometer which our search was based on (Fig. 3A–D). Additionally, the secondary structures of *tetR*, *σ*^*70*^, and the *agsA* thermometers all contain an SD sequence base paired with four uracil nucleotides, illustrating the accuracy of RNArobo in predicting secondary structure based on a handmade descriptor. Handcrafted variants of our *agsA* search can be designed to discover other similar fourU thermometers. Moreover, the secondary structure of our new thermometers can serve as a template for future searches.

### Validation of RNA thermometer activity

**FIGURE 4.**
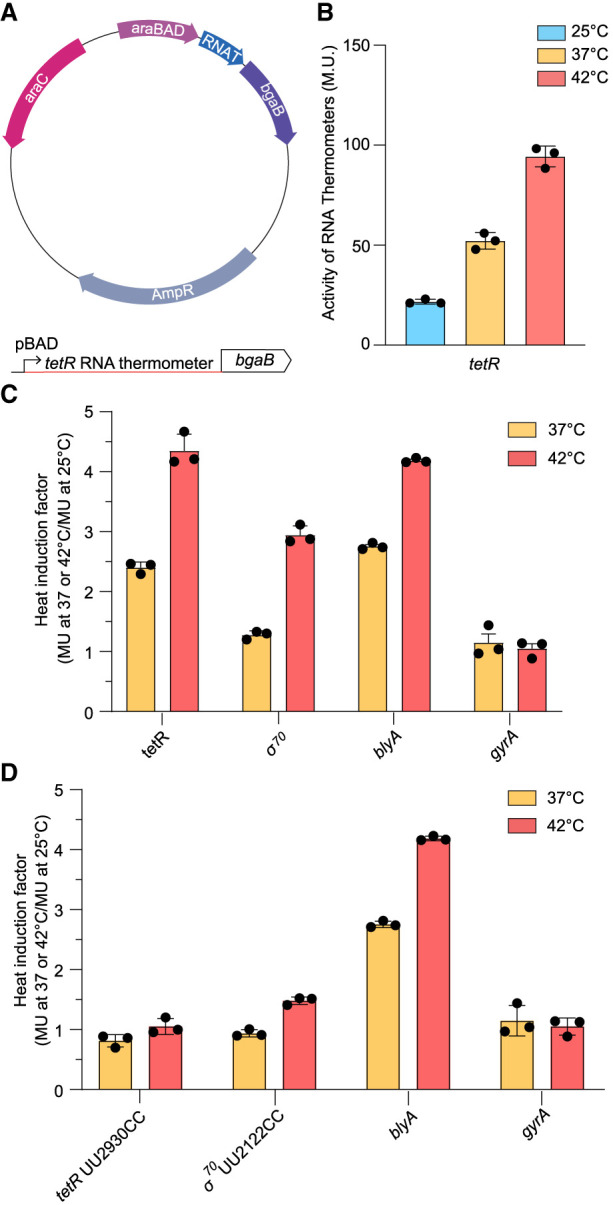
β-Galactosidase assays of the 5′-UTR–*bgaB* fusions. (*A*) The used inducible reporter plasmid with the candidate thermometer placed directly upstream of the thermal stable β-galactosidase gene (*bgaB*) along with a schematic of *bgaB* fusions. (*B*) The activity of the *tetR* thermometer is expressed in Miller units. The 5′-UTR–*bgaB* fusions were incubated at 25°C, 37°C, and 42°C in triplicates, and their absorbances were measured to determine the expression of *bgaB* at each temperature. The Miller units were calculated for all 5′-UTR–*bgaB* fusions using the absorbances, and can be seen in Supplemental Figure S2. (*C*) Heat induction profiles for 5′-UTR–*bgaB* fusions, with the expressions at 25°C, 37°C, and 42°C compared to a positive control, the *blyA* fourU thermometer, and a negative control, DNA gyrase (*gyrA*). (*D*) Heat induction profiles of the mutated RNA thermometers. The heat inductions of the UU2930CC *tetR* and the UU2122CC *σ*^*70*^ mutants were calculated, and compared to the *blyA* positive control and *gyrA* negative control.

Once candidate sequences were identified, the putative DNA sequences were cloned into a reporter plasmid, positioned directly upstream of a thermally stable β-galactosidase (*bgaB*) enzyme (Fig. 4A,B). Because of its stability under heat stress, *bgaB* is a reliable reporter that has been used to characterize several RNA thermometers (Hirata et al. 1984; Klinkert et al. 2012). The candidate RNA sequence replaces the native 5′-UTR of *bgaB* and contains the ribosome-binding site (RBS) sequence necessary for translation (Supplemental Table S2). Temperature-dependent expression was determined by heat induction of *E. coli* cells expressing the candidate 5′-UTR–*bgaB* fusions. The reporter plasmid contains a P_BAD_ promoter and transcription was induced by arabinose immediately before heat induction. Cells containing the *tetR* or *σ*^*70*^–*bgaB* fusions were incubated for 60 min at 25°C, 37°C, or 42°C to examine expression. Subsequently, β-galactosidase activity was measured for each temperature (Supplemental Fig. S2), and heat induction profiles were calculated for each RNA thermometer (activity in Miller units [M.U.] at 37°C/25°C or 42°C/25°C).

Heat shock of cells expressing *tetR*–*bgaB* fusion resulted in heat induction factors ∼2.4- and 4.3-fold at 37°C and 42°C, respectively. Interestingly, heat shock of cells expressing *σ*^*70*^–*bgaB* fusion resulted in no significant heat induction at 37°C (∼1.3). In contrast, at 42°C, the same *σ*^*70*^–*bgaB* fusion resulted in heat induction factors of approximately threefold (Fig. 4C). *bgaB* fusions containing the previously established *blyA* RNA thermometer (Tong et al. 2023) were used as positive controls. As a negative control, the 5′-UTR of a DNA gyrase gene (*gyrA*), which is not thermally regulated, was used. These findings demonstrate that the 5′-UTR of our candidates modulates reporter gene activity in a temperature-dependent manner, with a notable increase in the level of gene expression at increased temperatures.

To further validate that the candidate sequences tested were directly responsible for the thermal regulation of gene expression, mutations were made to strengthen and stabilize base-pairing. We used previously successful stabilizing mutations based on the predicted secondary structure (Waldminghaus et al. 2007b; Tong et al. 2023). For motifs based on the fourU thermometers, the strongest stabilizing mutations are made in the fourU region across from the SD sequence to change G•U wobble base pairs to stronger canonical G–C base pairs. Stabilizing mutations were made to the *tetR* (UU2930CC) and *σ*^*70*^ (UU2122CC) RNA thermometers and decreased thermometer activity to negative control levels (Fig. 4D). These mutations demonstrate that the noncanonical G•U wobble base pairs within the fourU motif are essential for temperature-sensitive gene regulation. Overall, our results indicate that thermoregulation of the *tetR* and *σ*^*70*^ RNA thermometers is directly dependent on the thermal stability of the fourU motif.

## DISCUSSION

### Future directions

The steps outlined in our results are sufficient for identifying potential RNA thermometer candidates and validating the thermoregulatory function of those candidates. In addition to validation of thermometer activity, an additional step to further characterize the system and gain insight into the mechanisms underlying thermoregulation is strongly suggested. We previously characterized the *blyA* thermometer through structure probing and gene expression experiments. Structure probing, such as selective 2′-hydroxyl acylation analyzed by primer extension (SHAPE) (Merino et al. 2005; Spitale et al. 2013), in-line probing (Regulski and Breaker 2008), and DMS probing (Wells et al. 2000; Rouskin et al. 2014), can be performed to profile changes in secondary structure at different temperatures. In our previous report (Tong et al. 2023), we used SHAPE to investigate conformational changes due to heat. SHAPE provides information on secondary structure at a single-nucleotide resolution. The reactivity of each RNA base is correlated with the flexibility of the 2′-OH, with single-stranded or flexible regions exhibiting increased reactivity in opposition to regions engaged in base-pairing or other interactions. Additionally, to investigate differences between transcriptional and translational control in the system, transcript levels of *bgaB* fusions should be measured by quantitative real-time PCR (qRT-PCR) at different temperatures. Cells for qRT-PCR should be harvested at the same time as β-galactosidase assays for consistent results. Northern blot analysis can also be used to measure RNA transcript levels (Böhme et al. 2012).

Gene-specific experiments should also be considered to gain knowledge of the particular thermoregulation being investigated. One such experiment particular to the *tetR* RNA thermometer discovered here would be the investigation of tetracycline resistance under different temperatures in *E. coli* and *S. flexneri*. Results would provide an additional layer of knowledge on how the *tetR* RNA thermometer affects bacterial survival in the presence of tetracycline under different temperatures. A prospective experiment specific to the *σ*^*70*^ thermometer would be to test if any genes with a *σ*^*70*^ promoter are up-regulated on the transcriptional level at higher temperatures in *M. gnavus* and *B. pectinophilus*. Both *tetR* and *σ*^*70*^ are vital genes for cell survival, and full investigations on how these newly reported thermometers alter gene function will be of great importance.

These investigations provide details on how RNA thermometers respond to heat stress on a structural and molecular level and will be applied to the *tetR* and *σ*^*70*^ thermometers in future studies. Other biophysical investigations, such as NMR studies, X-ray crystallography, circular dichroism, fluorescence studies, and thermal stability comparisons, can be used to complement the characterization of these temperature-sensitive RNA molecules.

### Application of Robo-Therm pipeline

Many naturally occurring RNA thermometers have been discovered and subsequently characterized; however, the extent to which these RNA thermometers occur remains unclear. We successfully developed Robo-Therm to identify and validate potential RNA thermometers across various genomes based on the highly customizable motif-driven program, RNArobo, and a well-established expression investigation based on the thermally stable β-galactosidase. Robo-Therm could be applied to any RNA thermometers with established secondary structures. From our Robo-Therm search, we identified two interesting candidates occurring in the 5′-UTR of different genes across different prokaryotic genomes. These new thermometers have a high predicted secondary structure homology with the RNA structure they were based on, exemplifying the utility of Robo-Therm in discovering RNA thermometers based significantly on a known structure. Furthermore, our pipeline outlines how to identify and incorporate sequence elements into important structural elements, which can be applied to any RNA thermometer search. These findings suggest that RNA thermometers may play an important role in antibiotic resistance and regulating gene expression in bacteria of the gut microbiome.

Thermosensitive regulation of virulence gene expression has been discovered in various bacteria (Loh et al. 2018), but our previous discovery of the *blyA* thermometer revealed only the second RNA thermometer in a phage genome (Altuvia et al. 1989; Tong et al. 2023), and the identification of the *σ*^*70*^ thermometer in bacteriophage Caudoviricetes implies that RNA thermometers might be more prevalent in bacteriophages than previously anticipated. The discovery of the *blyA* thermometer linked the previously described thermal regulation of *blyA* (Regamey and Karamata 1998) to the thermally regulated life cycle of the bacteriophage SPβ. The identity of the same *σ*^*70*^ thermometer in two prevalent gut bacteria and the gut bacteriophage Caudoviricetes proposes a direct phage–host relationship that may be regulated by heat. The role of the *σ*^*70*^ thermometer in these organisms should be investigated further. Additionally, thermometers in viruses have been found to function beyond modulating gene expression in their hosts. An RNA thermometer in the flavivirus West Nile controls flavivirus replication during host switching (Meyer et al. 2020). These studies suggest that RNA thermometers may be integral to the life cycles of many viruses.

To date, temperature-sensing RNA elements have not been fully explored in prokaryotes and eukaryotes. Our bioinformatics-based pipeline can be applied to investigate the occurrence of any RNA thermometer in different genomes to reveal the possible widespread occurrence of RNA thermometers. Robo-Therm integrates in silico approaches with direct experimentation in order to reveal the abundance of potential RNA thermometers across multiple genomes.

## MATERIALS AND METHODS

### RNA motif search in genomic sequences

Bacterial genomic sequences were downloaded from the NIH National Library of Medicine, National Center for Biotechnology Information, using the Nucleotide search (https://www.ncbi.nlm.nih.gov/nuccore/). RNArobo (Rampášek et al. 2016) was used to perform an RNA motif search using the following descriptor:

s1 h1 s2 h2 s3 h2′ s4 h1′ s5
s1 0  NNN******
h1 0:0 *NNNNN:NNNNN*
s2 0  N*
h2 0:0 *CUUUUNNNN*:*NNNNAGGAG*
s3 0  NNN****
s4 0  N*
s5 0  NAUG***

See the RNArobo article (Rampášek et al. 2016) for detailed instructions on descriptor search design. Results were manually curated and verified with the Basic Local Alignment Search Tool (BLAST) (https://blast.ncbi.nlm.nih.gov/).

### Plasmid construction

The initial plasmid backbone was synthesized from VectorBuilder (VectorBuilder Inc.). The 5′-UTR of thermometer candidates were placed directly upstream of a heat-stable β-galactosidase from *Bacillus stearothermophilus* and driven by a pBAD promoter (pBAD: β-galactosidase). ATG (start codon) in the thermometer sequences replaces the first ATG of *bgaB*. NEBuilder HiFi DNA Assembly was used to insert sequences into the same plasmid backbone described above. NEBuilder HiFi DNA Assembly was performed according to the manufacturer's protocol. A previously described vector used to validate the *blyA* thermometer (Tong et al. 2023) (VectorBuilder ID: VB220225-1020jdm, can be retrieved from https://en.vectorbuilder.com/design/retrieve.html) was used as the backbone for plasmid construction. NEBuilder Assembly Tool 2.0 was used to design fragments. Sequences of mutants for β-galactosidase assay (Supplemental Table S2) were designed with the following complementary flanking sequences to the VB220225-1020jdm plasmid.

5′-ATACCCGTTTTTTGGGCTAA—Sequences for β-galactosidase assay (Supplemental Table S2)—AATGTGTTATCCTCAATTTG-3′

### β-Galactosidase assays

*E. coli* DH5α cells carrying *bgaB* plasmids were grown overnight at 25°C in LB broth plus 100 μg/mL ampicillin. Overnight cultures were diluted in LB broth plus 100 μg/mL ampicillin to an optical density at 600 nm (OD600) of 0.1, and then grown at 25°C to an OD600 of 0.3–0.5. Transcription was induced with 0.01% (w/v) arabinose addition, then they were split and incubated at 25°C, 37°C, or 42°C. After 60 min, 500 μL samples were taken, OD600 was measured, and samples were used for β-galactosidase assays as previously described (Miller 1972; Zhang and Bremer 1995; Tong et al. 2023), with the following modifications. Three 20-μL samples of culture were added to 80 μL of permeabilization solution (0.8 mg/mL hexadecyltrimethylammonium bromide, 0.4 mg/mL sodium deoxycholate, 100 mM Na_2_HPO_4_, 20 mM KCl, 2 mM MgSO_4_, and 5.4 μL/mL β-mercaptoethanol). After a 30-min incubation at 30°C, 600 μL of substrate solution (60 mM Na_2_HPO_4_, 40 mM NaH_2_PO_4_, 1 mg/mL *o*-nitrophenyl-β-d-galactoside (ONPG), 2.7 μL/mL β-mercaptoethanol) was added. The reactions were incubated for 90 min at 55°C . The addition of 700 μL of 1 M Na_2_CO_3_ terminated the reactions to be prepared for absorbance readings. Assays were performed in triplicate. Heat induction factor was calculated by dividing expression in Miller units at 37°C or 42°C by expression at 25°C.

## SUPPLEMENTAL MATERIAL

Supplemental material is available for this article.

## Figures and Tables

**Maria T. Almanza:**
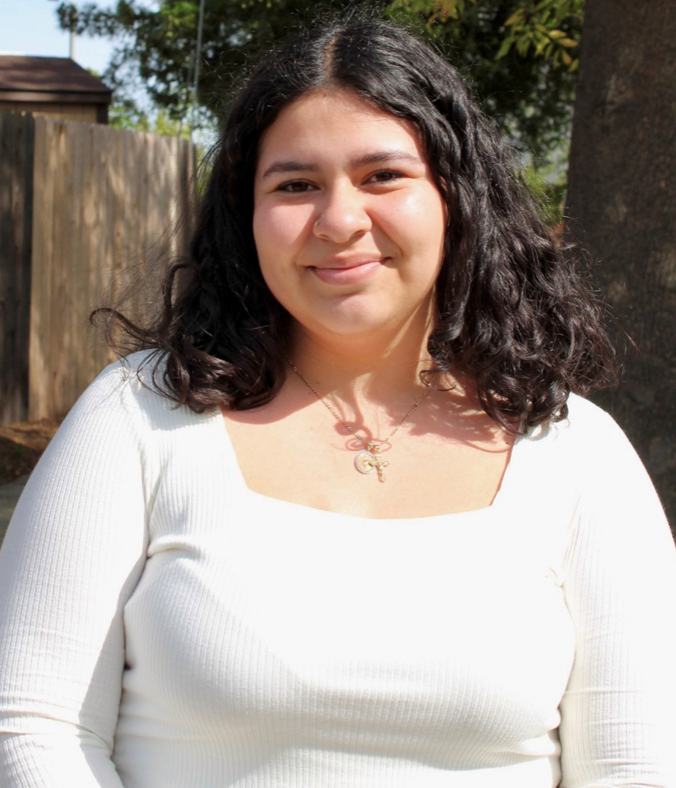


**Andrea V. Banks:**
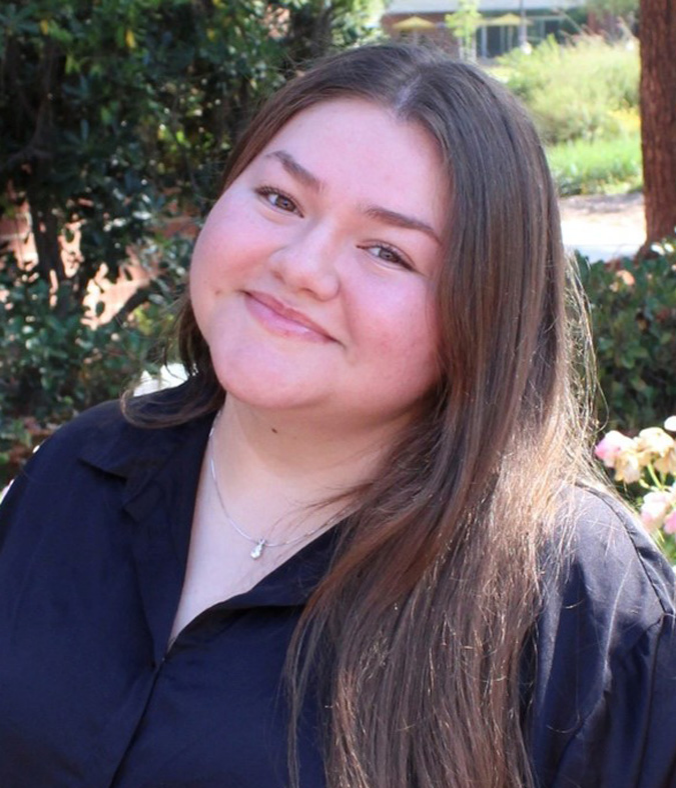


**Alyssa M. Castellanos:**
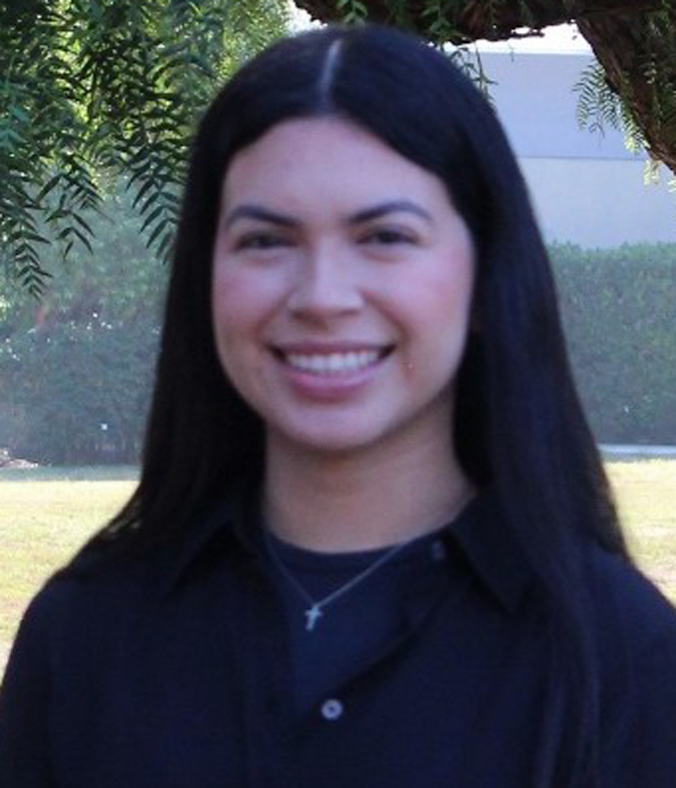


**Catherine G.O. Hernandez:**
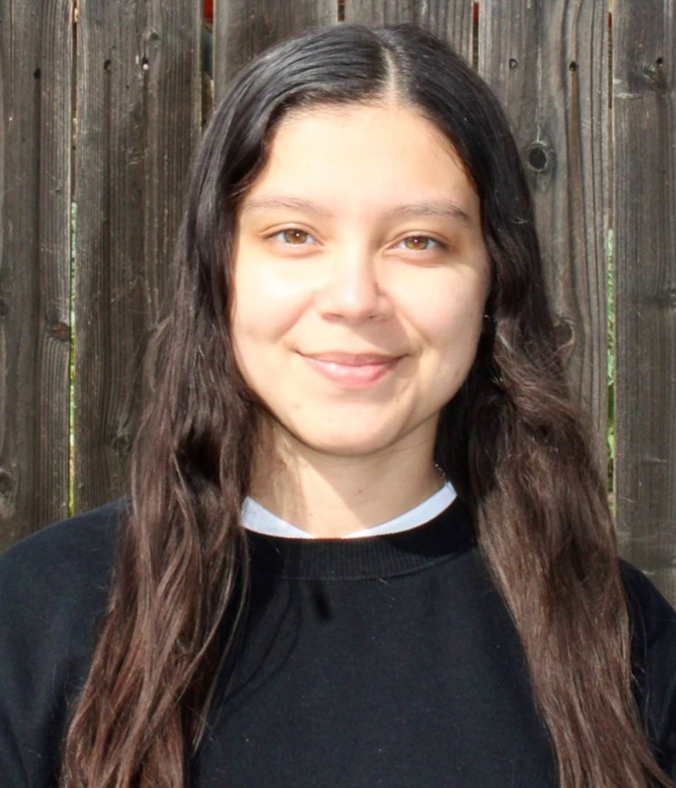


**Monica L. Lopez:**
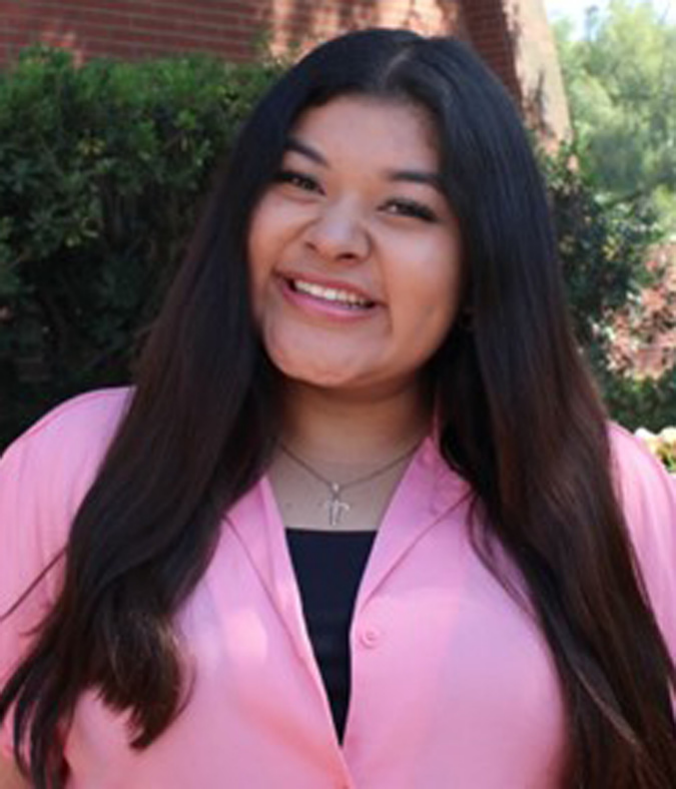


**Daniela Rodriguez:**
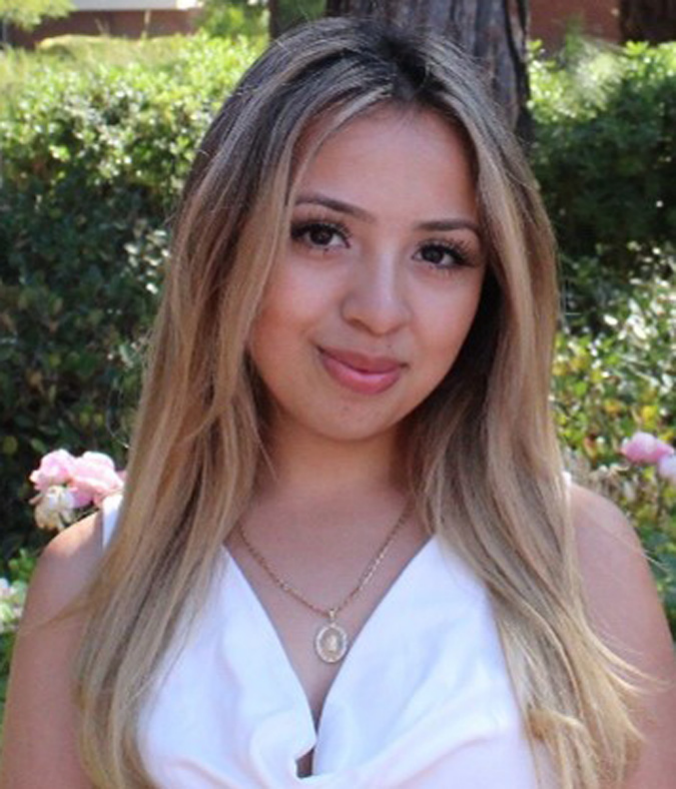


**Davis M. Sharts:**
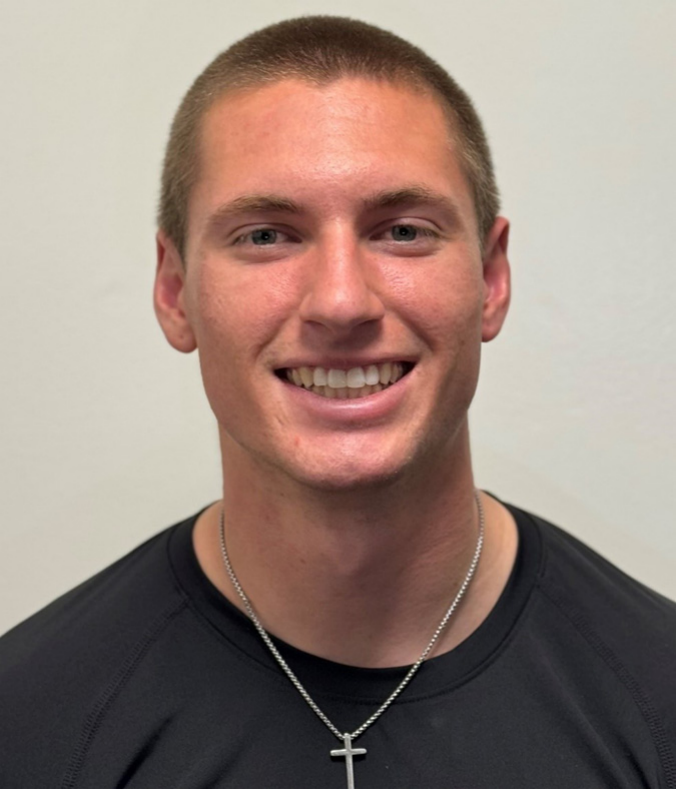


## MEET THE FIRST AUTHORS

***Meet the First Author(s)* is an editorial feature within *RNA*, in which the first author(s) of research-based papers in each issue have the opportunity to introduce themselves and their work to readers of *RNA* and the RNA research community. Davis M. Sharts, Maria T. Almanza, Andrea V. Banks, Alyssa M. Castellanos, Catherine G.O. Hernandez, Monica L. Lopez, and Daniela Rodriguez are co-first authors of this paper, “Robo-Therm, a pipeline to RNA thermometer discovery and validation.” This research was conducted at California Lutheran University, under the mentorship of Dr. Michael Abdelsayed, by a majority of first-generation Hispanic undergraduate students looking to further their education and scientific careers. The project was largely funded by the Ronald E. McNair Postbaccalaureate Achievement Program, which prepares first-generation college students from traditionally underrepresented groups for PhD programs by expanding their undergraduate experience.**

**What are the major results described in your paper and how do they impact this branch of the field?**

Our bioinformatic pipeline, Robo-Therm, combines an adaptive and user-friendly in silico motif search with an efficient reporter system to facilitate RNA thermometer discovery in bacterial and viral genomes. Through our application of Robo-Therm, we discovered two novel four U RNA thermometers in bacterial and bacteriophage genomes found in the human gut. One of these thermometers is present in the 5′-UTR of a gene that codes for a housekeeping *σ*^*70*^ RNA polymerase subunit in the bacteria *Mediterraneibacter gnavus* and *Bacteroides pectinophilus*, and in the bacteriophage Caudoviricetes, which infects *B. pectinophilus*. The other thermometer is in the 5′-UTR of a tetracycline resistance gene (*tetR*) in the intestinal bacteria *Escherichia coli* and *Shigella flexneri*. Our Robo-Therm pipeline is a powerful tool that can be applied to discover multiple RNA thermometers across various genomes, furthering this understudied area of science.

**What led you to study RNA or this aspect of RNA science?**

Once we began learning under the mentorship of Dr. Michael Abdelsayed, his passion for this niche area of molecular biology and biochemistry was contagious. Under his guidance, we understood the importance that RNA thermometers have on gene expression and that they are incredibly undervalued. As young scientists who are interested in molecular and biomedical sciences, we will carry our research with us as we begin our journeys into graduate school.

**During the course of these experiments, were there any surprising results or particular difficulties that altered your thinking and subsequent focus?**

Although the *tetR* thermometer adds fascinating insight for increasing antibiotic resistance in bacteria, the most surprising result was the validation of a thermometer in front of a *σ*^*70*^ RNA polymerase subunit gene. To our knowledge, this gene has not been proven to be thermally regulated by a functional RNA and, more interestingly, the *σ*^*70*^ thermometer exists in a bacteria and bacteriophage genome. Our discovery raises more questions as to whether other *σ* factor genes are thermally regulated by functional RNAs in bacteria. Our research also implies that RNA thermometers might be more prevalent in bacteriophages than previously anticipated.
